# Platelet-rich fibrin as a hemostatic agent in dental extractions in patients taking anticoagulants or antiplatelet medication: a systematic review

**DOI:** 10.1007/s00784-024-05983-x

**Published:** 2024-10-10

**Authors:** Marie Sophie Katz, Mark Ooms, Marius Heitzer, Timm Steiner, Anna Bock, Florian Peters, Frank Hölzle, Ali Modabber

**Affiliations:** Department of Oral and Maxillofacial Surgery, University Hospital RWTH Aachen, Pauwelsstraße 30, 52074 Aachen, Germany

**Keywords:** Platelet-rich fibrin, PRF, Hemostatic agent, Dental extraction, Hemostasis, Postoperative bleeding

## Abstract

**Objectives:**

The aim of this systematic review was to evaluate whether platelet-rich-fibrin (PRF) is effective in preventing postoperative bleeding after dental extractions in patients on anticoagulation or antiplatelet therapy compared to stitches alone and different hemostatic agents.

**Materials and methods:**

This systematic review was conducted and reported according to the Preferred Reporting Items for Systematic Reviews and Meta-Analyses (PRISMA). The protocol was registered at the International Prospective Register of Systematic Reviews (PROSPERO) (registration number CRD42024562289). Two authors independently performed searches in several databases, including PubMed, EMBASE, Cochrane Library, and SCOPUS.

**Results:**

In total, 789 studies were identified, of which 11 met the inclusion criteria after full-text screening. Four studies evaluated the efficiency of PRF in patients on antiplatelet therapy, and seven studies analyzed its hemostatic effect in patients on anticoagulants. All studies showed sufficient hemostasis when PRF was used, but due to heterogeneity meta-analysis was not possible.

**Conclusions:**

Despite the use of different protocols and control groups, PRF treatment seems to be superior to only stitches and inferior to chitosan dressings concerning the time of hemostasis. Additionally, PRF seems to be beneficial in terms of faster wound healing and less postoperative pain.

**Clinical relevance:**

PRF is known to enhance soft tissue healing and reduce postoperative pain. As a fully autologous platelet concentrate, it can support hemostasis after dental extractions in patients on antiplatelet or anticoagulation therapy. This systematic review aims to provide an update of the existing literature on PRF and its hemostatic capacity in patients with blood thinning medication.

## Objectives

 Platelet-rich fibrin (PRF) has become a versatile and widely used agent in dentistry and medicine [1–3]. Its properties for enhancing and supporting wound healing are suitable for and commonly used in socket and ridge preservation and periodontal treatments, and to minimize pain and postoperative discomfort in oral surgery [4–11].

Autologous platelet concentrates (APC) have a long history and have undergone an evolution from platelet-rich plasma (PRP) and platelet-rich in growth factors (PRGF) to PRF [12, 13].

Compared to other platelet products, Choukroun’s PRF, invented in 2001, is the only fully autologous fibrin without the addition of any anticoagulants, and it can be applied in several forms, such as liquid, gel, plugs, or membranes [14–16]. Its ability to coagulate is accompanied by a higher share and prolonged release of growth factors, such as TGF-ß1, VEGF, IL-1β, IGF-1, and PDGF-AB [17, 18]. It therefore combines all the advantages of a stable blood clot, just without the red blood cells attached.

The increasing share of patients taking anticoagulants or antiplatelet medication (AP) presents a challenge in oral surgery to provide treatment with a low risk of thromboembolic incidents, and on the other hand, ensure a low incidence of postoperative bleeding episodes [19, 20]. There is a strong trend to continue blood thinning medications during minor oral surgeries and to perform these procedures in outpatient settings [21, 22].

Various agents can be used as hemostatic plugs or dressings to induce hemostasis and prevent postoperative bleeding episodes: tranexamic acid, xenogeneic gelatin sponges or collagen, or chitosan dressings manufactured from freeze-dried shrimp shells [23–27]. Most of these are well tolerated, but since they are not fully autologous, foreign body reactions and allergies have been reported [28–30].

In the search for a fully autologous hemostatic material, there have been attempts to use PRF as a concentrate rich in thrombocytes [31, 32]. Based on which antithrombic medication the patient takes, coagulation is altered, so preparation protocols must be adapted as described by Marinho et al. [33].

While clotting of the PRF plug still works in patients on antithrombic medication, Ockerman et al. found that the PRF membranes of patients under oral anticoagulation seemed to be weaker and contain fewer leukocytes; however, patients on AP medication showed no difference from the control group not on any medication [34]. Concerning the macroscopic and microscopic fibrin architecture of PRF, Bootkrajang et al. found no difference between patients on warfarin and healthy controls [35].

The aim of this systematic review was to evaluate whether PRF is an effective hemostatic agent to prevent postoperative bleeding after dental extractions in patients under anticoagulation or AP therapy.

## Materials and methods

### Protocol development and eligibility criteria

This review was conducted and reported according to the Preferred Reporting Items for Systematic Reviews and Meta-Analyses (PRISMA) [36]. The protocol was registered at the International Prospective Register of Systematic Reviews (PROSPERO) with registration number CRD42024562289 [37].

A protocol including all aspects of a systematic review methodology was developed prior to the initiation of this review. This included the definition of a focused question, a PICOS (patient, intervention, comparison, outcome, and study design) question, a defined search strategy, study inclusion criteria, determination of outcome measures, screening methods, data extraction, and analysis, and data synthesis.

### Defining the focused question

The following focused question was defined: “Is PRF effective as a hemostatic agent in dental extractions in patients under antiplatelet or anticoagulation therapy?”

### PICOS question

P: Among patients taking anticoagulation or antiplatelet therapy undergoing dental extractions.
I: Does the use of PRF as a hemostatic agent.
C: When compared to other hemostatic agents or control sites.
O: Result in changes in hemostasis, bleeding, and postoperative pain.
S: Clinical studies in humans.

### Search strategy

Two authors (MSK and AM) independently performed an electronic search in several databases, including PubMed, EMBASE, Cochrane Library, and SCOPUS. Articles published up to June 1st, 2024, were considered. No language or time restrictions were applied in the search.

### Search terms

The electronic search strategy used the following combination of key words: (“hemostasis” OR “haemostasis” OR “hemostatic” OR “haemostatic” OR “Dental Extraction” OR “Extraction” OR “Tooth removal” OR “Teeth removal” OR “postoperative bleeding”) AND (“Leukocyte platelet-rich-fibrin” OR “platelet-rich-fibrin” OR “LPRF” OR “L-PRF” OR “Advanced platelet-rich-fibrin” OR “APRF” OR “A-PRF” OR “A-PRF+”). Additionally, the reference lists of review articles and the articles included in the present review were screened.

### Study selection and inclusion criteria

The study selection criteria were studies in German or English. Only clinical studies in humans using autologous PRF were included. Studies using other platelet concentrates, such as PRP or PRGF, were excluded, as were studies evaluating the hemostatic effect of PRF in patients not taking anticoagulants or antiplatelet medication.

### Screening and selection of studies

The titles and abstracts of the selected studies were independently screened by two reviewers (MSK and AM) based on the question, “Is PRF effective as a hemostatic agent in dental extractions in patients under antiplatelet or anticoagulation therapy?” Discrepancies were solved by discussion between two authors (MSK and AM) and a judge (MO). Cohen’s Kappa coefficient was calculated as a measure of agreement between the two reviewers. Subsequently, full-text articles were obtained if the answer to the screening was “yes” or “uncertain.”

### Data extraction and analysis

The following data were extracted: author(s), year of publication, type of study, number of patients, treatment and control groups, type of medication, primary outcome measurement, and significance value. All studies were classified according to the study design to provide an overview of all studies matching the search criteria. Afterwards, the outcomes were compared in separate tables and discussed. Due to heterogeneity of the study protocols a comprehensive statistical analysis of their outcomes was not possible.

## Results

### Selection of studies

**Fig. 1:**
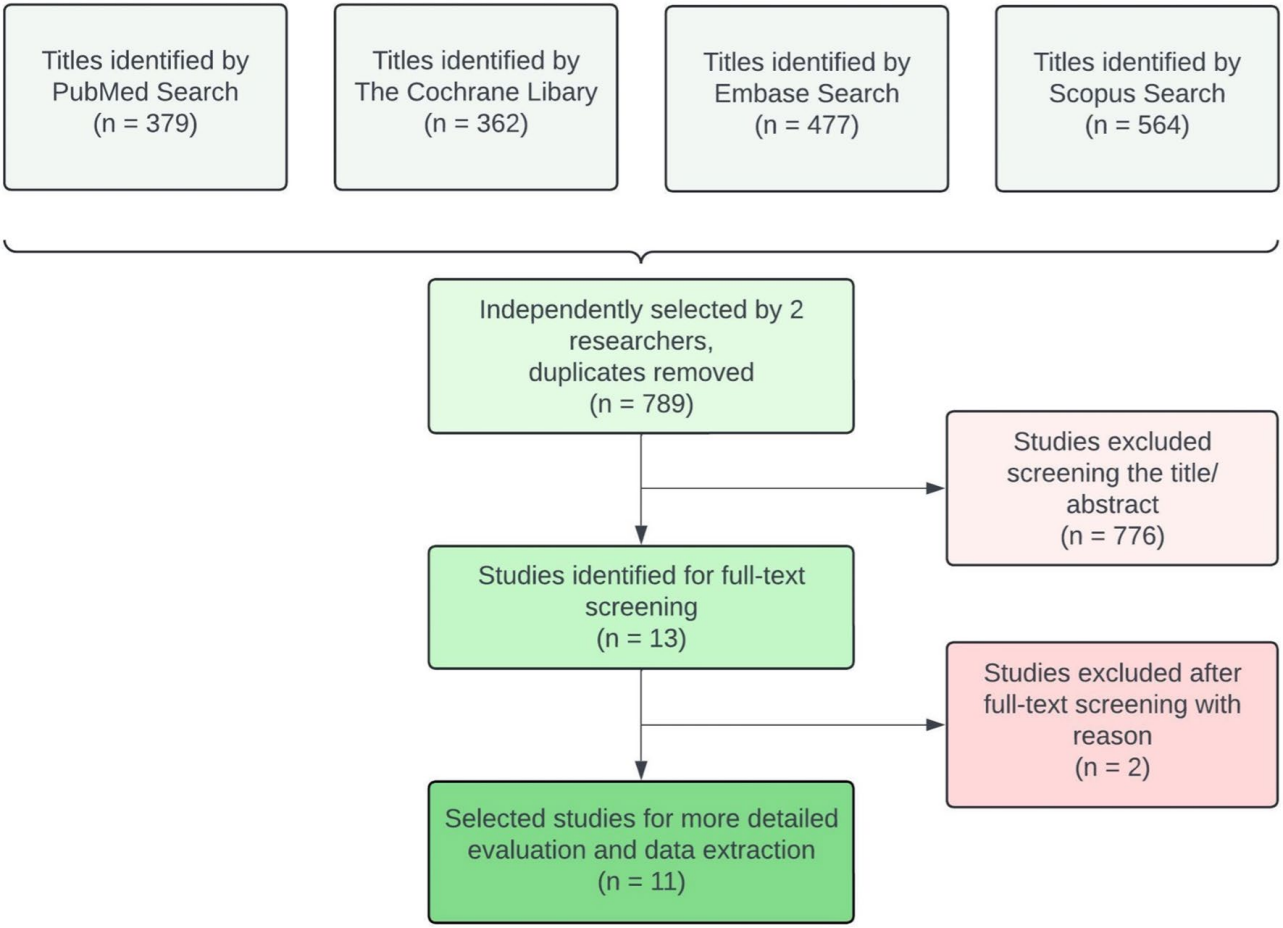
Flow diagram of study identification, screening and inclusion process adapted from PRISMA

The database searches identified 1782 articles, and after removing duplicates, 789 remained (Fig. 1). After the two independent researchers screened the titles and abstracts, 13 were found to meet the inclusion criteria and were selected for full-text analysis (inter-reviewer agreement κ = 0.822). After detailed evaluation, two articles were dismissed based on the exclusion criteria, as they used PRF as a hemostatic agent but not in patients taking anticoagulants or AP medication [31, 32]. Finally, 11 studies were considered relevant and were included in this systematic review.

### Study characteristics

Classifying the types of studies included, there were three clinical studies without a control group compared to PRF: Sammartino et al. [38], de Almeida Barros Mourão et al. [39], and Berton et al. [40]. One study by Harfoush et al. [41] was a controlled clinical study without randomization, and seven studies were randomized clinical trials (RCTs): Eldibany et al. [42], Sarkar et al. [43], Giudice et al. [44], Munawar et al. [45], Brancaccio et al. [46], Rajendra et al. [47], and Kyyak et al. [48]. Of these, two were split-mouth studies [44, 46], and only one was blinded [48]. The patient cohorts included ranged from 20 to 300 patients, with a total number of 864 patients and at least 1148 teeth extracted. PRF was compared to dry gauze, chitosan, hemostatic plugs, gelatin sponges, tranexamic acid, and control sites with only stitches (Table 1).

**Table 1:** Study characteristics and patient cohorts

Authors (Year)	Type of study	Number of patients	Number of teeth extracted	Groups
Sammartino et al. (2011) [38]	Prospective clinical single-arm study	50 (20 males, 20 females; mean age 54.5 years)	168 (75 maxillary, 93 mandibular extractions; no patient had more than 4 teeth extracted)	L-PRF (no control group)
Eldibany et al (2014) [42]	Prospective RCT	20 (11 males [55%] and 9 females [45%]; mean age 46.65 years)	20 teeth (mandibular posterior teeth)	PRF + chitosan dressing
Harfoush et al. (2016) [41]	Prospective clinical controlled study	50 (34 males, 16 females; mean age 63 years)	50 (no information about maxilla/mandible, anterior/posterior)	PRF + dry gauze
de Almeida Barros Mourão et al. (2018) [39]	Prospective cohort study	25 (10 males, 15 females; mean age 72.44 years)	44 (24 mandibular, 20 maxillary teeth extractions)	PRF (no control group)
Sarkar et al. (2019) [43]	Prospective RCT	60 (29 males, 31 females; mean age 58.77 years)	60 (no information about maxilla/mandible, anterior/posterior)	PRF + chitosan
Giudice et al. (2019) [44]	Prospective RCT	40 split mouth design (28 males, 12 females; mean age 60.9 years);	160 (4 teeth per patient were extracted)	Control (just stitches) + hemostatic cellulose plug + L-PRF + A-PRF+
Munawar et al. (2020) [45]	Prospective RCT	84 (50 = 59.8% males and 34 = 40.5% females; aged 30–70 years)	84	PRF + tranexamic acid
Brancaccio et al.(2021) [46]	Prospective RCT	102 split mouth design (74 males, 28 females; mean age 64.1 years)	At least 4 teeth per patient were extracted; no information about exact number	Control (just stitches) + hemostatic cellulose plug + L-PRF + A-PRF+
Rajendra et al.(2021) [47]	Prospective RCT	300 (aged 35–70 years; no information about the share of males and females or mean age)	300	PRF + chitosan dressing
Berton et al.(2022) [40]	Prospective cohort study	112 (66 males, 46 females; mean age 77.1 years)	112 (all removed by easy extraction in < 15 min; 53 teeth single-rooted; 59 teeth multi-rooted)	L-PRF (no control group)
Kyyak et al. (2023) [48]	Prospective double-blind RCT	21 split mouth design (10 males, 11 females; mean age 71.04)	42 (no osteotomies; 22 maxillas; 20 mandibles; 22 anterior teeth; 20 posterior teeth)	A-PRF + gelatin sponge

*RCT *randomized clinical trial,* PRF *platelet-rich fibrin,* L-PRF *leukocyte-platelet-rich fibrin,* A-PRF *advanced platelet-rich fibrin

Four studies included patients under AP medication, four studies involved patients taking vitamin K antagonists (VKA), and three studies included patients under direct oral anticoagulants (DOAC). One study did not include any information about the anticoagulant (Table 2). The PRF protocols varied from Leukocyte-platelet-rich fibrin (L-PRF) protocols with normal or prolonged centrifugation time [38, 40, 44, 46] to Advanced platelet-rich fibrin (A-PRF/A-PRF+) [44, 46, 48], and in six studies, the preparation protocol or type of PRF was not further classified [39, 41–43, 45, 47].

**Table 2:** Comparison of platelet-rich fibrin (PRF) protocols, medication, and outcomes of the included studies

Author (Year)	PRF type	PRF protocol	Medication	Main outcome	*p*-value
Sammartino et al. (2011)[38]	L-PRF	3000 rpm for 18 min	Warfarin, (mean international normalized ratio [INR] 3.16)	Two postoperative bleeding episodes in patients with an INR of 3.7, which could be solved with local compression of the wound; 20% had mild postoperative bleeding. No cases of alveolitis.	–
Eldibany et al. (2014) [42]	PRF, not further classified	3000 rpm for 12 min	Warfarin, INR ≤ 3.5 (mean INR 2.28)	Both groups showed no delayed bleeding and complete hemostasis. Patients with chitosan showed more alveolitis, delayed healing, and greater pain.	–
Harfoush et al. (2016) [41]	PRF, not further classified	Not classified	Warfarin, INR ≤ 3.5 (mean INR 2.4)	Bleeding duration > 20 min was more frequent in the control group than in the PRF group (pain was not assessed).	*p* < 0.001
de Almeida Barros Mourão (2018) [39]	PRF, not further classified	12 min centrifugation; rpm not classified	DOAC (21x rivaroxaban; 4x apixaban)	No bleeding events at 24, 48, and 72 h after procedure; no complications.	–
Sarkar et al. (2019) [43]	PRF, not further classified	3000 rpm for 10 min	Antiplatelet	Hemostasis was faster in the chitosan group (mean time 1.182 min) than in the PRF group (mean time 2.64 min). Healing score after 7 days was better in the PRF group. Pain score was better in the PRF group (but no information about p-values).	*p* < 0.001 for hemostasis *p* < 0.001 for wound healing
Giudice et al.(2019) [44]	A-PRF + and L-PRF	For A-PRF+: 1300 rpm for 8 min For L-PRF: 2700 rpm for 18 min	Antiplatelet (29x acetylsalicylic acid [ASA]; 5x clopidogrel; 2x ticagrelor; 2x ASA + clopidogrel; 2x: ASA + ticagrelor)	A-PRF + showed significantly less bleeding after 30 min than the control group. All bleeding cases were moderate. No significant difference in patient preferences. No significant difference concerning wound healing index after 1 and 2 weeks.	*p* < 0.0361 for bleeding between the A-PRF + and control groups; *p* = 0.633 for wound healing index after 1 week and *p* = 0.255 for wound healing index after 2 weeks.
Munawar et al. (2020) [45]	PRF, not further classified	3000 rpm for 12 min	Anticoagulant (no further information; INR ≤ 3.5)	PRF group: 1/41 case of bleeding. Tranexamic acid group: 3/39 cases of bleeding. No significant difference.	*p* = 0.306
Brancaccio et al. (2021) [46]	A-PRF + and L-PRF	For A-PRF+: 1300 rpm for 8 min For L-PRF: 2700 rpm for 18 min	Antiplatelet (72x [70%] ASA; 15 [15%] clopidogrel; 5 [5%] ticagrelor; 6 [6%]: ASA + clopidogrel; 4 [4%: ASA + ticagrelor])	Less bleeding in the A-PRF + and L-PRF group. L-PRF showed less incomplete healing than the control group.	*p* < 0.05 for bleeding compared between the A-PRF + and control groups; *p* < 0.05 for bleeding compared between A-PRF + and hemostatic cellulose plug groups; *p* < 0.05 for bleeding compared between L-PRF group and control groups; *p* < 0.05 for wound healing compared between L-PRF and control groups.
Rajendra et al. (2021) [47]	PRF, not further classified	3000 rpm for 10 min	Antiplatelet	Hemostasis faster in the chitosan group (1.25 ± 0.06 min) than in the PRF group (1.89 ± 0.54 min). No statistically significant difference concerning postoperative pain.	*p* < 0.001 for hemostasis; *p* = 0.8 for postoperative pain.
Berton et al.(2022) [40]	L-PRF	2700 rpm for 18 min	53x VKA (51x warfarin; 2x acenocoumarol; mean INR 2.4) + 59x DOAC (15x dabigatran; 17x rivarixaban; 17x apixaban; 11x edoxaban)	Postoperative bleeding in 17% of VKA patients and 15.3% of DOAC patients. All mild bleeding that could be solved by local compression.	*p* = 0.31 for bleeding episodes in VKA patients compared with DOAC patients
Kyyak et al.(2023) [48]	A-PRF	1200 rpm for 8 min	DOAC (9x rivaroxaban; 7x apixaban; 5x edoxaban)	67% of mild oozing which could be stopped after 30–90 min by compression. No significant difference between the PRF group and gelatin sponge group.	*p* > 0.05 for bleeding events.

*PRF* platelet-rich fibrin, *L-PRF* leukocyte-platelet-rich fibrin, *A-PRF*  advanced platelet-rich fibrin, *INR* international normalized ratio, *ASA * acetylsalicylic acid, *DOAC* direct oral anticoagulant

All studies reported only mild to moderate bleeding events that could mostly be solved by local compression. PRF showed superior results for hemostasis compared to dry gauze [41], cellulose [46], and stitches only [44, 46].

In other studies, there were no significant differences between PRF and a chitosan dressing [42], tranexamic acid [45], or gelatin [48] concerning postoperative bleeding episodes. Sarkar et al. and Rajendra et al. found that hemostasis was faster in the chitosan group than in the PRF group [43, 47].

### Risk of bias (RoB) in the individual studies

The selected studies were individually screened using version 2 of the Cochrane tool for risk of bias in randomized trials (RoB 2). Five showed a low RoB, and six presented a moderate RoB (Table 3).

**Table 3:**
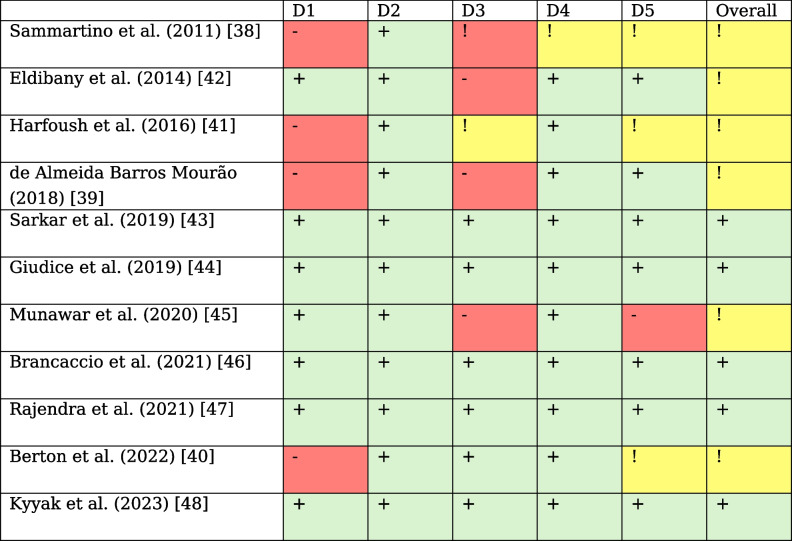
Individual bias of each included study based on the RoB 2 tool

*D1* randomization process, *D2* deviations from the intended interventions, *D3* missing outcome data, *D4* measurement of the outcome, *D5* selection of the reported result

**Fig. 2:**
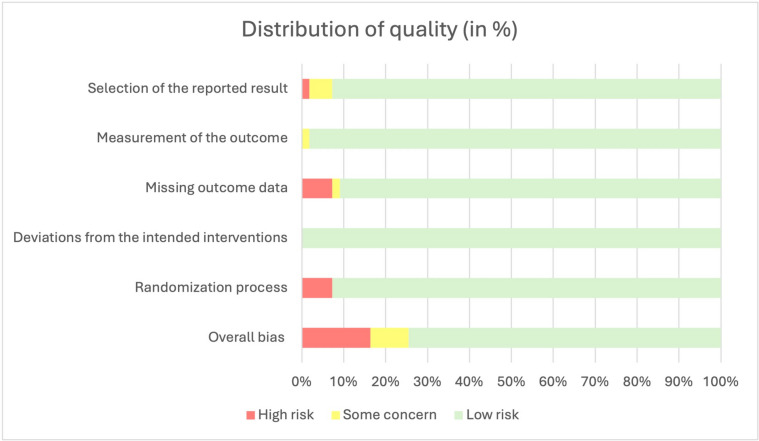
Distribution of quality assessments among the included studies (based on the RoB 2 tool)

The overall quality was good, and most concerns were due to a lack of randomization and the extent of the outcome data (Fig. 2).

## Discussion

There is a growing range of uses for PRF in all fields of dentistry, and keeping reasonable and evidence-based indications in perspective can be challenging. The aim of this review was to evaluate whether PRF is an effective hemostatic agent in dental extractions in patients under antiplatelet or anticoagulation therapy. To our knowledge, there have been two former reviews looking into this topic, one by Filho et al. that included three studies in 2021 [49], and one by Campana et al. that summarized six studies but included different forms of APCs (PRF and PRP) [50]. Since the number of studies using PRF as a hemostatic agent has increased, a contemporary look at the results of further investigations is needed for a knowledge update.

Different forms of medication bring different risks for postoperative bleeding episodes with them but can also alter the fibrin clotting process [34]. Some studies have adapted their PRF protocols to a longer centrifugation time to adjust for patients taking VKA [38, 40] or AP medication [44, 46].

Overall, PRF derived from any protocol seemed to be feasible as a hemostatic agent, since the bleeding incidents described were all moderate and could be handled by compression. Although the studies by Sarkar et al. [43] and Rajendra et al. [47] that investigated patients taking AP therapy found faster hemostasis in wounds with chitosan compared to PRF, the bleeding was stabilized in 2–3 min in both studies, which is still a favorable result. In the study by Eldibany et al., none of the groups (chitosan vs. PRF) showed delayed bleeding, but the patients treated with chitosan showed more alveolitis, delayed healing, and greater pain [42]. Likewise, Sarkar et al. found better wound healing and less pain in the PRF group compared to the chitosan group, which presents PRF as slightly inferior in time to bleeding control, but superior in patient comfort and cost [43].

Giudice at al. [44], Munawar et al. [45], Harfoush et al. [41], and Brancaccio et al. [46], who also evaluated the use of PRF in patients on antiplatelet found significantly less bleeding in sockets with A-PRF + than in control sockets with only stitches [44, 46], dry gauze [41], or a control group with tranexamic acid [45]. Nevertheless, it must be stated that the risk for postoperative bleeding is low overall in patients under AP monotherapy [51].

Patients taking VKA or DOAC have a higher incidence of postoperative bleeding after dental extractions [52, 53]. Sammartino et al., Eldibany et al., and Harfoush et al. evaluated the use of PRF in patients taking warfarin [38, 42]. In Sammartino et al.’s study, two postoperative bleeding episodes occurred in patients with an INR of 3.7 (mean INR 3.16), which could be solved with local compression of the wound [38]. Eldibany et al. reported no delayed bleeding at all, but the mean INR was lower in this study cohort (mean INR 2.28), which is a critical factor according to Febbo et al., who found a significantly higher risk for postoperative hemorrhage in patients with an INR ≥ 3 [54]. The study by Harfoush et al. found a higher incidence of bleeding > 20 min in patients without PRF compared to the PRF group in a study cohort with a mean INR of 2.4 [41]. Although the INR value seems to be the most important parameter in bleeding control after dental extractions, all three studies showed that PRF might be an additional benefit in stabilizing the coagulum and with its hemostatic effect.

Looking at studies using PRF in patients under DOAC, de Almeida et al. found no bleeding incidents overall [39], while Kyyak et al. [48] and Berton et al. (2022) [40] described mild oozing, which could be managed by compression. Of these three studies, only Kyyak et al. compared the PRF group to a control group (gelatin sponge), finding no significant difference between the groups [48]. As only one randomized clinical trial has suggested that PRF is not inferior to the use of gelatin as a hemostatic agent, the effect of PRF in DOAC patients is difficult to anticipate, and more studies with a control group are needed.

Additionally, the number and type of extractions, as well as mucosal incisions, also have an influence on the postoperative bleeding risk [55]. None of the studies included in this review analyzed correlations between extractions, osteotomies, and anterior or posterior extractions with bleeding outcomes, which is an important limitation concerning comparability.

Overall, it is not easy to retrace the isolated effect of PRF on postoperative hemorrhage. Only three studies compared the use of PRF with a control group treated with stitches or compression; three compared it to chitosan, one to tranexamic acid, and one to a gelatin sponge. Three studies did not include a control group. Moreover, the studies used different protocols for the preparation of PRF. This effect might be marginal concerning postoperative bleeding episodes, since all PRF protocols lead to coagulation and hence resemble stable blood clots. However, the amount of growth factor and its effect on wound healing may have differed. At least two studies, by Giudice at al. [44] and Brancaccio et al. [46], compared A-PRF to L-PRF. Giudice et al. found that A-PRF was superior to stitches alone concerning postoperative bleeding and wound healing after one and two weeks [44]. In contrast, Brancaccio et al. found similar bleeding rates in the L-PRF and A-PRF groups but better wound healing in the L-PRF group [46].

Due to heterogeneity of the studies a comprehensive statistical analysis of their outcomes was not possible, which must be addressed as a limitation of this review. Future randomized studies with a standardized PRF protocol comparing PRF to control sites and to different hemostatic agents should be performed. It is important to address the kind of medication and the extent of the operation (e.g., anterior or posterior teeth, extractions or osteotomies) to make studies comparable and to conclude the hemostatic potential of PRF in relation to other agents.

## Conclusion

PRF is known to enhance soft tissue healing and reduce postoperative pain. As a fully autologous platelet concentrate, a PRF clot can also support hemostasis after dental extractions in patients taking antiplatelet or anticoagulation therapy. Despite the use of different protocols and control groups, PRF treatment seems to be superior to only stitches and inferior to chitosan dressings concerning the time of hemostasis. Still, randomized clinical studies comparing PRF as a hemostatic agent to a control group are lacking, and further research evaluating the use of PRF in the context of the extent of the extraction is needed.

## Data Availability

No datasets were generated or analysed during the current study.

## Abbreviations

*PRF*: Platelet-rich fibrin
*APC*: Autologous platelet concentrates
*PRP*: Platelet-rich plasma
*PRGF*: Platelet-rich in growth factors
*AP*: Antiplatelet
*RCT*: Randomized clinical trial
*VKA*: Vitamin K antagonists
*DOAC*: Direct oral anticoagulants
*RoB*: Risk of bias
